# Evaluation of gel and structural properties of pork myofibrillar protein gels using faba bean protein isolate at various salt concentrations

**DOI:** 10.5713/ab.250921

**Published:** 2026-05-07

**Authors:** Geon Ho Kim, Koo Bok Chin

**Affiliations:** 1 Department of Animal Science, Chonnam National University, Gwangju, Korea

**Keywords:** Faba Bean Protein Isolate, Gelation, Pork Myofibrillar Protein, Salt Concentration, Structural Properties

## Abstract

**Objective:**

This study aimed to evaluate the gel and structural properties of pork myofibrillar protein gels (MPGs) using faba bean protein isolate (FBPI) at various salt concentrations.

**Methods:**

MPGs were prepared with or without 1 % FBPI under three salt concentrations (0.15, 0.30, and 0.45 M), yielding six treatments. The gel strength, viscosity, and cooking yield were measured. In addition, microstructure, surface hydrophobicity, sulfhydryl group, and sodium dodecyl sulfate-polyacrylamide gel electrophoresis were analyzed to examine the effects of FBPI and salt concentration on the gel and structural properties of MPGs.

**Results:**

MPGs containing 1.0% FBPI had a higher cooking yield, gel strength, protein surface hydrophobicity, and shear stress values than the control groups. The addition of FBPI resulted in more homogenous MPGs with a denser surface compared to the control groups. No interactions in functional properties between FBPI addition and salt concentration were observed, except for gel strength. MPGs at a 0.45 M salt concentration showed a higher cooking yield and hydrophobicity and fewer sulfhydryl groups than those at a 0.15 M salt concentration. The shear stress values at a 0.45 M salt concentration dramatically increased compared to those at 0.15 M and 0.30 M. Furthermore, higher salt concentrations (0.45 M salt) resulted in fewer cavities and a more compact three-dimensional structure.

**Conclusion:**

Higher salt concentrations may improve the rheological properties of MPGs and influence the effects of FBPI on the viscosity and protein patterns. These findings suggested that controlling salt concentration and FBPI level could be an effective strategy to enhance the gelation and structural characteristics of meat protein systems.

## INTRODUCTION

Myofibrillar proteins (MP) are one of the groups constituting skeletal muscle proteins and contribute to determining the type of muscle fiber [1]. MP contribute to the functional properties of meat products, such as water binding, emulsification, and gelation [2]. Moreover, MP is responsible for the quality (e.g., textural properties) and processing yield of meat products [3]. In addition, MP solubilization influences the quality of processed meats by forming interactions between the protein and water via hydrogen bonds and is markedly affected by ionic strength. The solubility of MP increases with an increase in the ionic strength of salt due to the depolymerization of myosin molecules in thick filaments into monomers; thus, ionic strength is the most important factor affecting the solubilization and functional properties of muscle proteins [4]. Thus, the effects of various ionic strength of MP on functional ingredients should be considered for developing desirable final products for consumers. These findings indicate that the control of salt concentration is critical for improving the extraction and functional properties of MPs in processed meats.

Legume proteins are often used as binders or extenders for reducing the fat content and improving the sensory properties of meat-based products [5]. Faba bean (*Vicia faba* L.), also called broad bean, fava bean, or field bean, has a high nutritional value as it has a higher protein-to-carbohydrate ratio (approximately 55.2%) than pea (45.0%) and chickpea (33.6%), and contains functional ingredients such as vitamins, fiber, and minerals. Thus, faba bean is considered as a sustainable protein source [6]. Hence, faba bean can enhance the stability and structure of processed foods, and shows potential for innovative and healthier foods [7]. In current trends, the utilization of legume proteins as functional ingredients has expanded, and they have been investigated as potential functional agents in maintaining texture and yield in meat protein systems.

Several studies have been conducted on the application of legume proteins to MPs, and have reported an improvement in the rheological and functional properties of the products. Jang and Chin [8] reported that pork MP with red bean protein isolate exhibited an increased cooking yield (CY) and shear stress. In a study by Kim and Chin [9], the addition of chickpea powder into pork MP improved functional and gelling properties compared to the control group. Wu et al. [10] reported that kidney bean protein could be applied to chicken MP to enhance cross-linking and the functional properties of the protein. These reports suggested that legume proteins can interact synergistically with MPs, resulting in improving their gelation and water-holding capacity (WHC) under appropriate conditions.

Faba bean protein has been reported to have functional properties comparable to soy protein, which is already widely used in food and meat systems, supporting its potential as an alternative protein ingredient [11,12]. In addition, faba bean protein differs from soy protein in that it is non-allergic and non-genetically modified [13]. Accordingly, faba bean protein has considerable potential as a functional agent in meat protein systems. Nevertheless, the functional properties of pork MP with faba bean protein may also be sensitive to the ionic strength of the formulation. Langton et al. [14] reported that faba bean proteins extracted at different salt concentrations had different rheological properties and microstructures. These findings indicated that ionic strength of salt affected the performance of faba bean protein as an extender and binder in meat products. However, few studies have evaluated the rheological and functional properties of purified salt-soluble proteins incorporated with faba bean proteins under various salt concentrations. The effect of ionic strength on the interaction between faba bean protein and pork MP is important for understanding and optimizing gelation and structural stability in meat protein systems with varying salt concentrations. Therefore, we evaluated the gel and structural properties of pork MP gels (MPGs) with faba bean protein isolate (FBPI) at different salt (NaCl, sodium chloride) concentrations, providing a basis for the development of high-quality meat products.

## MATERIALS AND METHODS

### Materials

Pork loin (Landrace×Yorkshire×Duroc crossbred castrated pig) was used as raw meat for extracting MP. The external fat and connective tissue on the surface of the raw meat were removed and the meat was cut into cubes (1–2 cm^3^). The meat was then packaged in polyethylene/nylon bags under vacuum and stored in a freezer at −20°C. FBPI with a protein concentration of approximately 90% was supplied by Yantai Shuangta Food.

### Preparation of pork myofibrillar protein gel

Frozen pork loin cubes were thawed in a refrigerator at 4°C for 12 h. The meat was then homogenized with 0.1 M sodium chloride and 50 mM sodium phosphate buffer (pH 6.25) for 90 s. Next, the homogenate was centrifuged (Supra 22; Hanil Scientific) for 15 min at 1,660×g. After removing the supernatant, the precipitate was collected, homogenized with the buffer solution, and centrifuged again. This process was performed in triplicate. The precipitate was filtered with 0.1 M sodium chloride buffer for removing connective tissue, and centrifuged. The protein content of MP was measured using the Biuret method, and MP was adjusted to a protein concentration of 4%. Based on the previous study by Kim and Chin [15], the FBPI level was fixed at 1.0%. At a 4% MP concentration, 1.0% FBPI was used to provide a measurable functional contribution without excessively altering the base MP matrix. The experimental design is shown in Table 1. The salt concentrations of the control and treatment groups were adjusted by adding a buffer with 0.15 M, 0.30 M, and 0.45 M NaCl at pH 6.50 to investigate the rheological properties of MPGs at different salt concentrations.

### Cooking yield

The sample was heated in a glass tube placed in a water bath (WB-22; Daihan Scientific) at 20°C–80°C (rate 2°C/min). The sample was weighed after cooling for 12 h at 4°C and stored at room temperature for 30 min to remove moisture exudate. The CY was calculated by substituting the measured weight of the sample into the formula below:


(1) $$\text{Cooking yield }(\%)=\frac{\text{Weight of the sample after heating }(\text{g})}{\text{Weight of the sample before heating }(\text{g})}\times 100$$

### Gel strength

The gel strengths (GSs) of cooked MPGs were determined using an Instron Universal Testing Machine (Model 3344; Instron) with the Merlin software (Instron). The first peak of the breaking force (gf) was recorded as the GS by single-cycle compression with a probe at a speed of 500 mm/min.

### Viscosity

Unheated MPGs were used for viscosity measurements. The viscosity was recorded as the change in shear stress (Pa) with an increasing shear rate (1/s) ranging from 0 to 600/s. A concentric cylinder-type rotational rheometer (RC30; RheoTec Messtechnik) was used. Excel (ver. 2016; Microsoft) was used to plot a graph of the viscosity results.

### Protein surface hydrophobicity

The protein surface hydrophobicity (H_0_) was determined using the method of Chelh et al. [16] The protein concentration was adjusted to 10 mg/mL with 20 mM sodium phosphate buffer (pH 6.0). Subsequently, 1 mL of the mixture was blended with 200 μL of 1 mg/mL bromophenol blue (BPB) solution. A control without MPG was prepared with 1 mL of phosphate buffer and 200 μL of BPB solution. The samples and control were centrifuged at 2,000×g for 15 min. The optical density (OD) values of the samples and control were measured with a spectrophotometer (X-ma 1200; Human Corporation) set at 595 nm, and were used to calculate the H_0_ values by applying the formula below:


(2) $${\text{H}}_{0}\;(\mu \text{g})=200\;\mu \text{L}\times (\text{OD of control}-\text{OD of sample})/(\text{OD of control})$$

### Sulfhydryl group

A modified Ellman’s method [17] was used to measure sulfhydryl (SH) groups (μmol/g). Samples were prepared by mixing 0.1 g of MPG, 8.4 mL of double distilled water, 0.5 mL of 4 mg/mL 5,5′-dithiobis-2-nitrobenzoic acid solution, and 1 mL of Tris-Gly-8M Urea. After mixing, the samples were stored at room temperature for 5 min. The OD values were measured at a wavelength of 412 nm.

### Sodium dodecyl sulfate-polyacrylamide gel electrophoresis

Sodium dodecyl sulfate-polyacrylamide gel electrophoresis (SDS-PAGE) was performed to investigate the effects of various salt concentrations on the polymerization of MPGs with FBPI. Samples were adjusted to a 10 mg/mL protein concentration and loaded on a 10% acrylamide separating gel (0.375 M Tris, pH 8.8) and 4% stacking gel (0.125 M Tris, pH 6.8) with Laemmli sample buffer (Cat. Number 161-0737; Bio-Rad Laboratories). Electrophoresis was run at 150 V for 1.5 h to separate the protein bands. A standard marker (Cat. Number 161-0318; Bio-Rad Laboratories) was used to determine the molecular weights of unknown proteins in the samples.

### Microstructure

The three-dimensional (3D) microstructure of MPG was detected using a low-vacuum scanning electron microscope (JSM-6610LV; JEOL). The samples were cut into a cuboidal shape (3 mm^3^) and soaked in 2.5% glutaraldehyde in 0.1 M sodium phosphate buffer (pH 7) for 24 h at 4°C for fixing. Subsequently, the samples were immersed in osmium tetroxide (OsO_4_) in 0.1 M sodium phosphate buffer (pH 7.00) for 5 h. Then, the samples were dehydrated using various concentrations (50%, 60%, 70%, 80%, 90%, and 100%) of ethanol solution and acetone for 10 min each. Dehydrated samples were gold-coated using an auto-sputter coater (Cressington Scientific Instruments). The microstructure of each sample was observed at a fixed magnification of 1,000×.

### Statistical analysis

All parameters were determined three times. Experimental data were expressed as the mean±standard deviation. Two-way (3 concentrations of salt×2 concentrations of FBPI) analysis of variance (ANOVA) was conducted using IBM SPSS Statistics 27 (IBM). If there were no interactions between the two factors of each parameter, data were pooled by the FBPI addition or salt concentrations. A post-hoc analysis was performed at p*<*0.05, using Duncan’s multiple range test. In addition, the heatmap was generated in MetaboAnalyst (ver. 6.0) to visualize interrelationships among the measured parameters such as CY, GS, H_0_, and SH group. Data were log_10_-transformed and clustered using Euclidean distance with complete linkage.

## RESULTS AND DISCUSSION

### Cooking yield, hydrophobicity and sulfhydryl group

Since there were no interactions between the FBPI addition and salt concentrations, the results of CY, H_0_, and SH group were pooled by FBPI addition and salt concentration as shown in Table 2. The CYs of the MPGs containing FBPI were higher than those of the control groups (p<0.05). The addition of FBPI decreased the moisture loss of MPGs during the heating process, suggested that FBPI enhanced the WHC of the heat-induced protein structure. Li et al. [18] reported that the addition of 2% or 4% soy protein isolate increased the CY of pork MPs compared to the control. Niu et al. [19] reported that MPGs with 0.5% or 0.75% soy protein isolate showed lower CLs than MPG alone (p<0.05), regardless of the pH. The enhanced hydrogen bonding with disulfide linkages and hydrophobic association contributes to the decrease in the CL of protein gels [20]. Moreover, faba beans contain legumin A and B (11S globulin), and these subunits might play a crucial role in hydrogen bonding and hydrophobic interactions between the protein networks by forming disulfide bonds [5,21]. MPG with 0.15 M salt showed the lowest CY among all salt concentrations evaluated, whereas MPG with 0.45 M salt had lower moisture loss than the samples with lower salt concentrations during cooking (p<0.05). Overall, the CYs of MPGs increased with increasing salt concentration. Chin et al. [22] reported CL of pork MPGs with 0.60 M salt was reduced compared to samples containing lower salt concentrations. This suggests that ionic strength influences the solubilization of protein by promoting water binding and gelation [23]. Thus, the WHC of MPGs improved with an increase in FBPI and salt concentration. Additionally, these results indicated that FBPI and salt produced similar functional effects on water retention.

As shown in Table 2, the H_0_ values of the control groups were lower than those of MPGs with FBPI (p<0.05). MPG with 0.45 M salt showed the highest *H_0_* values, whereas those of MPG with 0.15 M salt were the lowest H_0_ values (p<0.05). Moreover, an upward trend in the H_0_ values of MPGs was observed with increasing salt concentration. These results proved that the incorporation of FBPI into MPGs increased hydrophobicity as the salt concentration increased. These increases in H_0_ were attributed to partial unfolding of the protein matrix, which exposed hydrophobic residues, and facilitated intermolecular associations. H_0_ is a crucial indicator of structural changes in the protein resulting from interfacial behavior [24]. The increase in H_0_ observed with both FBPI addition and salt increase suggested that FBPI showed a comparable influence to salt on protein unfolding and hydrophobic exposure. Faba bean contains legumin (11S globulin), which has a dense structure because of strong hydrophobic interactions; thus, the protein shows high surface hydrophobicity [25,26]. Thus, FBPI can strengthen the protein structure by increasing hydrophobicity. Lee and Chin [27] reported that MPGs at higher salt concentrations showed greater hydrophobicity than those at lower salt concentrations. These authors concluded that a protein structure with high hydrophobicity enhances the WHC. Kang et al. [28] reported that, as the salt content increases, the unfolded structure is enhanced, and the hydrophobicity of MP increases by exposing amino acids and hydrophobic residues. Accordingly, the present study found that treatment groups that incorporated FBPI or higher salt concentrations resulted increased CYs.

Table 2 shows the SH groups of MPGs at different FBPI and salt concentrations. The control groups showed a higher content of SH groups than the FBPI treatments (p<0.05). This result suggested that legume proteins tended to be decreased SH groups in the MP structures. MPGs with 0.15 M salt had more SH groups than 0.30 M or 0.45 M salt (p<0.05). These results indicated that FBPI promoted the formation of disulfide bonding, thereby contributing a more compact MP network. The marked exposure of SH groups at 0.45 M suggested enhanced disulfide bond formation and stabilization of protein network. The exposure of SH groups enhances the MP structure by its 3D changes to the surface of molecules [29]. Both salt concentration and FBPI addition showed a decreasing trend in SH groups, suggesting that comparable disulfide bonding contributed to the stabilization of the protein network. In this study, the addition of FBPI decreased the number of SH groups and resulted in enhanced rheological properties because the 7S and 11S globulins contained faba bean reacted with amino acids such as cysteine and glutamine to form intramolecular disulfide bonding, which contributed to form the protein structure [30]. Lee and Chin [31] reported that the SH groups of MPGs decreased with increasing salt concentration regardless of the addition of gelatin, which was in agreement with the results of this study. An increase in salt concentration leads to an increase in protein solubility, and protein unfolding results in the exposure of buried SH groups [28]. Our results were similar to this trend and indicated that incorporating FBPI with the salt concentrations contributed to a stable protein structure. Additionally, the increase in H_0_ and the reduction in SH groups were associated with the improvement in GS (Table 3) and the formation of a more compact protein structure, indicating the involvement of reactive hydrophobic residues and disulfide bonds in gel development.

### Gel strength

Table 3 presents the GSs of MPGs with FBPI at different salt concentrations. The interactions between FBPI and salt concentration contributed to the increase in GS (p<0.05). Moreover, the GSs of MPGs with FBPI were higher than those of the control groups at all salt concentrations (p<0.05). These results indicated that the addition of FBPI to MPGs improved their GS. The increase in GS is associated with enhanced intermolecular cross-linking and a denser gel matrix through interactions, particularly under moderate salt concentrations as a result of balanced protein solubilization and aggregation. In general, legume proteins show the potential for improving functional properties such as the GS and WHC of the protein structure. Lee et al. [32] reported that MPGs that incorporated 1% or 2% red bean protein isolate had higher GSs than the control in the presence of microbial transglutaminase (MTGase). Li et al. [18] reported that the hardness of pork MPs increased with increasing soy protein isolate concentration. Storage proteins contained in legumes (e.g., conglycinin and glycinin) play a role in gelation and structural integrity via intramolecular disulfide bonding [30]. The GS increased with increased salt concentration (p<0.05), and FBPI increased GS at each salt level (p<0.05); moreover, the interactions between the presence of FBPI and salt concentration indicated that the effect of FBPI was strengthened at higher salt levels. Hong and Chin [33] reported that MPs with 0.3 M salt had higher GSs than treatments with lower salt concentrations (0.1 M or 0.2 M), regardless of the presence of MTGase. In addition, Sun and Holley [34] reported that the hardness values of pork MP with 0.4 M NaCl were lower than those of samples with 0.5 to 0.7 M NaCl. Salt is an essential factor in the formation of MP as it develops functional properties by solubilizing the protein. Furthermore, the GS of myosin depends on the salt concentration [35].

### Viscosity

**Figure 1:**
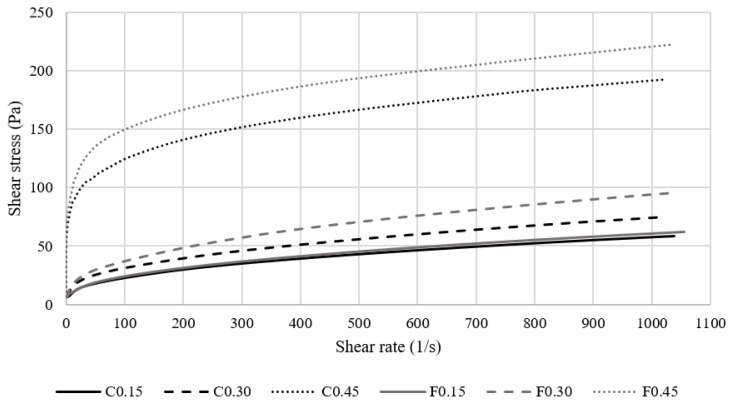
Viscosity of pork myofibrillar protein gels with faba bean protein isolate (FBPI) as affected by different salt levels. Treatments: C0.15, myofibrillar protein gel (MPG) with 0.15 M salt; C0.30, MPG with 0.30 M salt; C0.45, MPG with 0.45 M salt; F0.15, MPG with 0.15 M salt and 1.0% FBPI; F0.30, MPG with 0.30 M and 1.0% FBPI; F0.45, MPG with 0.45 M salt and 1.0% FBPI.

The shear stress values of MPGs with FBPI at different salt concentrations are presented in Figure 1. Regardless of the presence of FBPI, MPGs at a 0.45 M salt concentration had dramatically higher shear stress values than those at lower salt concentration. Moreover, the shear stress values of the samples with 0.30 M salt were slightly higher than those with 0.15 M salt. As the salt concentration decreased, viscosity decreased, regardless of the presence of FBPI. Zhu et al. [35] reported that the viscosity of chicken MP emulsions increased as the salt concentrations increased (0.2–0.6 M). They concluded that increased salt concentrations enhanced protein solubility and emulsion stability, leading to higher viscosity of MP emulsions. Furthermore, the MPG with FBPI and a 0.15 M salt concentration had a similar shear stress values to those of the control, whereas the shear stress values of the FBPI treatments were higher than those of the control groups when 0.30 M or 0.45 M salt was added. These results suggested that the presence of FBPI increased the viscosity of MPGs at salt concentrations above 0.30 M. Mohanan et al. [36] reported that faba bean protein concentrate increased the viscosity of the aqueous phase by enhancing interfacial membrane formation. Moreover, Langton et al. [14] reported that the rheological properties (e.g., viscosity and gelation) of faba bean protein are strongly influenced by the salt concentration because of the change in solubility of 7S globulins. Hence, the incorporation of FBPI increased the viscosity of MPGs, and this effect was greater at higher salt concentrations. These results suggested that the increase in viscosity was related to the improvement in GS (Table 3) in the presence of FBPI and at higher salt levels, indicating enhanced protein–protein interactions.

### Sodium dodecyl sulfate-polyacrylamide gel electrophoresis

**Figure 2:**
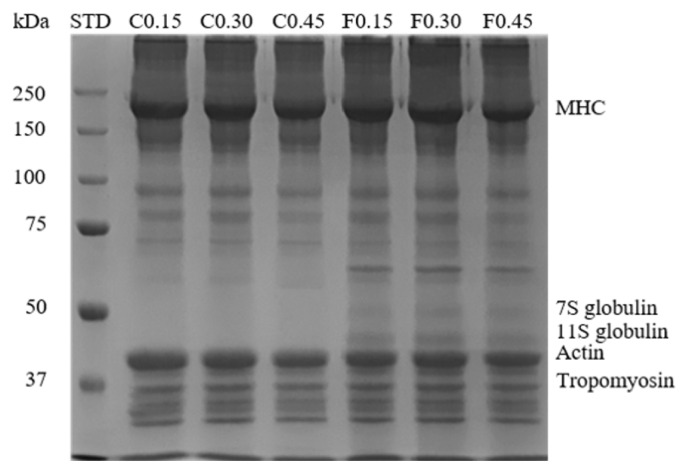
Sodium dodecyl sulfate polyacrylamide gel electrophoresis of myofibrillar protein gels (MPGs) with faba bean protein isolate (FBPI) as affected by different salt levels. Treatments: C0.15, MPG with 0.15 M salt; C0.30, MPG with 0.30 M salt; C0.45, MPG with 0.45 M salt; F0.15, MPG with 0.15 M salt and 1.0% FBPI; F0.30, MPG with 0.30 M and 1.0% FBPI; F0.45, MPG with 0.45 M salt and 1.0% FBPI. MHC, myosin heavy chain.

Figure 2 shows the protein profiles of MPGs with FBPI at different salt concentrations. The myosin heavy chain (MHC) bands of MPGs at 0.15 and 0.30 M salt concentrations were similar. By contrast, MPGs at a 0.45 M salt concentration had thinner MHC bands than those at lower salt concentrations, regardless of the presence of FBPI. Lee et al. [32] reported that the protein patterns of pork MPGs at 0.30 and 0.45 M salt concentrations showed MHC bands with reduced intensity compared to those at a 0.15 M concentration. The reduction in the MHC band intensity was due to the formation of large aggregates and is associated with an increase in protein surface hydrophobicity, resulting in an enhanced protein structure [37]. Some bands were observed in only FBPI added samples at 43 KDa and 50 KDa. These bands might be protein patterns corresponding to vicilin (7S globulin) and legumin (11S globulin). Bühler et al. [38] detected vicilin and legumin at 46–55 KDa and 38–40 KDa, respectively, in the faba bean protein concentrate profile. A characteristic band appeared at 62 KDa in the lanes of samples containing FBPI and was assumed to represent an interaction between FBPI and MP. The presence of globulins from legume proteins have the potential for enhancing GS through interactions with myosin [39]. In the FBPI-added sample at a 0.45 M salt concentration, the globulin intensity decreased compared to samples at lower salt concentrations (0.15 M and 0.30 M). These results were due to the strong association between the salt-solubility of globulins and changes in the protein structure [10]. Thus, the protein patterns of MPGs were structurally modified by the addition of FBPI and the different salt concentrations. The changes in SDS-PAGE profiles and apparent viscosity (Figure 1) reflected the alterations in protein conformation induced by the addition of FBPI and varying ionic strength, as reflected in SH groups and H_0_ values.

### Microstructure

**Figure 3:**
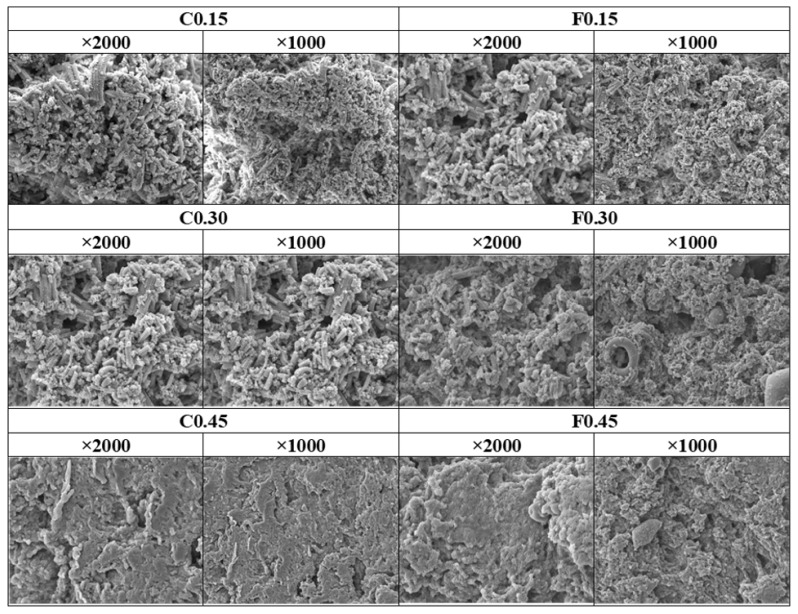
Microstructure of myofibrillar protein gel (MPG) added with faba bean protein isolate (FBPI) as affected by different salt levels. Treatments: C0.15, MPG with 0.15 M salt; C0.30, MPG with 0.30 M salt; C0.45, MPG with 0.45 M salt; F0.15, MPG with 0.15 M salt and 1.0% FBPI; F0.30, MPG with 0.30 M and 1.0% FBPI; F0.45, MPG with 0.45 M salt and 1.0% FBPI.

The microstructures of MPGs with FBPI at different salt concentrations are shown in Figure 3. The different salt concentrations modified 3D structure of the protein. MPGs at a 0.45 M salt concentration had more homogenous particles and a compact surface compared to those at lower salt concentrations regardless of FBPI addition. An increase in salt concentration resulted in a decrease in porosity and cavities on the surface of the MPGs. According to Sun and Holley [34], MPs at higher ionic strength formed adequate gels with salt solubilization and showed a coarsely aggregated structure in contrast to with lower ionic strength, which has a fine-stranded structure. In a micrograph of MPGs, no differences were observed between the control and FBPI treatment at a 0.15 M salt concentration. By contrast, the incorporation of FBPI resulted in flatter, more homogenous surface microstructures in MPGs at 0.30 and 0.45 M salt concentrations. These results might be related to the differences in viscosity between the control and the FBPI treatment, which increased dramatically when 0.30 and 0.45 M salt were used as compared to 0.15 M. In several studies, incorporating various legume proteins had improved the microstructure of the protein matrix. For instance, Lin et al. [40] reported that MPGs with soy protein isolate had smoother and denser microstructures than those without plant proteins as a result of the stronger association and gelation properties derived from the globulin subunits. Kudre et al. [41] reported that the addition of black bean and mung bean protein isolate to sardine surimi resulted in a more compact and ordered structure compared to the control. The formation of a more compact and continuous network structure was consistent with the improvement in GS (Table 3), and with changes in viscosity (Figure 1), H_0_, and SH groups (Table 2), indicating that modified protein–protein interactions and disulfide formation contributed to the protein network. Hence, adding FBPI and higher salt concentrations might change the protein matrix into a more stable structure because of the synergistic effect.

### Correlation of between the addition of faba bean protein isolate and salt concentration

**Figure 4:**
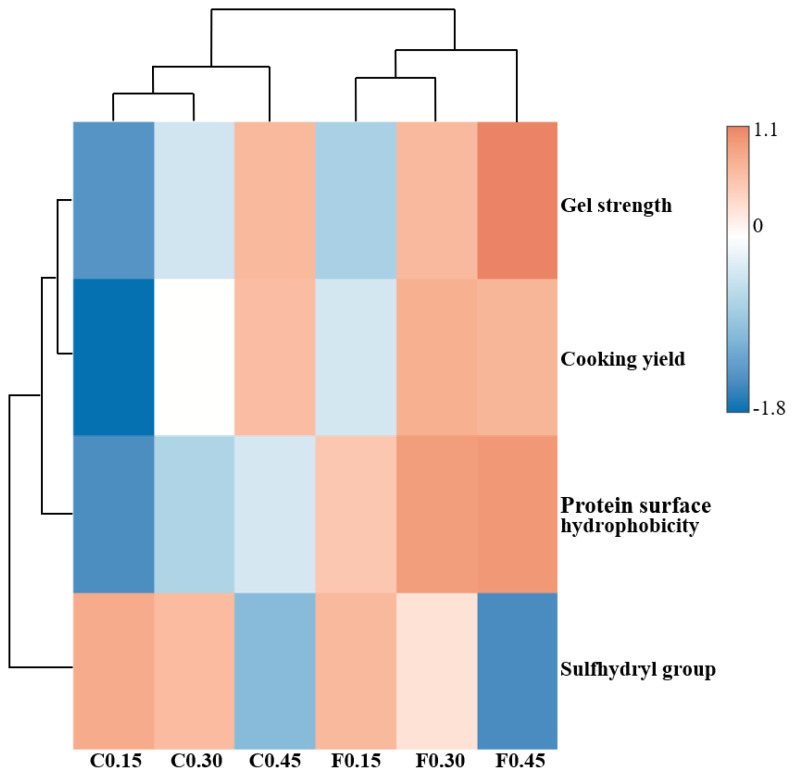
Heatmap of the correlation between the addition of faba bean protein isolate (FBPI) and salt concentration. Treatments: C0.15, myofibrillar protein gel (MPG) with 0.15 M salt; C0.30, MPG with 0.30 M salt; C0.45, MPG with 0.45 M salt; F0.15, MPG with 0.15 M salt and 1.0% FBPI; F0.30, MPG with 0.30 M and 1.0% FBPI; F0.45, MPG with 0.45 M salt and 1.0% FBPI. The color scale represents the direction of the correlation, showing red for positive and blue for negative relationships.

As shown in Figure 4, the correlation analysis generally supported the trends observed in the individual measurements. The positive correlation between CY and GS indicated that improved water retention was associated with stronger gel formation. In addition, the positive association between H_0_ and GS suggested that the exposure of hydrophobic residues was related to the development of a denser protein network. In contrast, SH groups showed an opposite trend, exhibiting a negative correlation with GS and H_0_, suggested that the reduction of total SH groups was associated with protein unfolding and enhanced disulfide bond formation. These relationships reflect structural changes associated with gel development, rather than independent effects of individual factors. These correlation patterns indicated that FBPI and salt acted synergistically to stabilize the protein network, confirming that the effectiveness of FBPI in improving functional properties was dependent on the ionic strength of the system.

## CONCLUSION

The addition of 1% FBPI improved the gel and structural properties of MPG, including the CY, GS, and viscosity, regardless of the salt concentration used. FBPI-added samples had higher H_0_ values and fewer SH groups than the control groups, and thus resulted in a modified protein structure. MPGs at higher salt concentrations showed a higher CY and GS than those at lower salt concentrations (0.45 M>0.30 M>0.15 M). Furthermore, MPGs with FBPI at high salt concentrations presented lower band intensities for MHC and globulins in the protein profiles, as well as microstructures with larger aggregates and lower porosity. In summary, the addition of FBPI improved gel and structural properties of pork MPGs by modifying the protein network and enhancing water retention, and its incorporation into the meat protein system was influenced by salt concentration. Moreover, FBPI exhibited functional trends similar to salt, increasing CY and H_0_, but reducing SH groups. Therefore, FBPI could be utilized as a functional ingredient to improve texture and yield in reduced-salt formulation, with potential application in meat processing.

## Figures and Tables

**Table 1:** Experimental design of pork myofibrillar protein gels with faba bean protein isolate (FBPI) at different salt

Ingredients (mg/mL)	Salt concentration (M)					

	Control			FBPI		
	
	0.15 M	0.30 M	0.45 M	0.15 M	0.30 M	0.45 M
Myofibrillar protein mixture	80.0	80.0	80.0	80.0	80.0	80.0
Buffer solution	20.0	20.0	20.0	19.0	19.0	19.0
FBPI	-	-	-	1.00	1.00	1.00
Total	100.0	100.0	100.0	100.0	100.0	100.0

**Table 2:** Cooking yield, protein surface hydrophobicity (H_0_), and sulfhydryl groups of myofibrillar protein gels with faba bean protein isolate at different salt concentrations

	Cooking yield (%)	H_0_ (μg)	Sulfhydryl groups (μmol/g)
Treatments			
CTL	73.6±20.1^b^	24.5±3.32^b^	24.3±1.85^a^
TRT	95.4±2.62^a^	35.6±1.73^a^	20.4±1.39^b^
Salt concentration (M)			
0.15	60.6±15.2^c^	27.2±7.26^b^	31.4±2.25^a^
0.30	86.9±1.07^b^	30.9±6.56^a^	26.6±3.77^b^
0.45	93.2±4.07^a^	32.0±5.89^a^	9.06±2.03^c^

a–c Means having the same superscripts in the same column are not different (p>0.05).

**Table 3:** Gel strength of myofibrillar protein gels with faba bean protein isolate (FBPI) at different salt concentrations

Treatments	Salt concentration (M)		

	0.15	0.30	0.45
Control	77.8±4.29^Bc^	124±15.8^Bb^	207±3.40^Ba^
FBPI	107±6.02^Ac^	206±8.17^Ab^	253±14.3^Aa^

A,B Means having the same superscripts in the same column are not different (p>0.05).

a–c Means having the same superscripts in the same row are not different (p>0.05).

## Data Availability

Upon reasonable request, the datasets of this study can be available from the corresponding author.
